# The Need for Integrating Governance, Operations, and Social Dynamics into Water Supply/Distribution Modelling^†^

**DOI:** 10.3390/engproc2024069012

**Published:** 2024-08-28

**Authors:** Lindell Ormsbee, Diana Byrne, Nicholas Magliocca

**Affiliations:** 1 Department of Civil Engineering, University of Kentucky, Lexington, KY 40506, USA;; 2 Department of Geography and the Environment, University of Alabama, Tuscaloosa, AL 35487, USA;

**Keywords:** ABMs, network models, sustainability, system dynamics

## Abstract

Water systems in the US are experiencing increasing challenges because of poor governance, unsustainable fiscal policies, an aging workforce, new environmental regulations, and concerns over environmental justice. These challenges will only increase if the specific constraints and barriers to system viability are not first identified and then translated into new policies and best management practices to ensure system sustainability, reliability, resilience, and equity of services. This paper proposes a methodology to accomplish this objective that integrates agent-based models, water distribution models, and sustainability performance models within a larger system dynamics framework.

## 1. Introduction

Water systems in the US are experiencing increasing challenges because of poor governance, unsustainable fiscal policies, an aging workforce, aging infrastructure, new environmental regulations, and concerns over environmental justice. This has led to several catastrophic failures in many economically distressed communities such as Flint, MI; Jackson, MS; Prichard, AL; and various rural communities in Appalachia, such as Martin County, KY. Such failures can only be expected to increase if the specific constraints and barriers to system viability are not first identified and then translated into new policies and best management practices to ensure system sustainability, reliability, resilience, and equity of services in response to new customer expectations and new stressors such as climate change.

While engineers and planners have used mathematical models of physical water infrastructure (e.g., EPANET [1]) to help guide the design and operation of such systems, such work has been traditionally done in isolation from the governance, operations, and social structures that typically influence, if not control, the ultimate sustainability of such systems. This paper will explore the use of a conceptual framework that integrates agent-based modelling, physical infrastructure modelling, performance modeling, and different stress ensembles (including climate scenarios) to examine both their episodic and long-term impacts on the performance and sustainability of rural water systems.

The impact of governance, operations, and social dynamics on water system viability will be explored using three different types of agents: governance agents (representing governing and funding agencies such as local water boards and state agencies), operational agents (representing the managers and operators of the local water utility), and social agents (representing the various classes of water users). The behavior and responses of these different agents to various stress scenarios (e.g., climate events, component failures, environmental regulations, increasing water rates, etc.) and other agent responses are explored using agent-based modelling and EPANET (for modelling impacts on the water distribution system). The developed model can then be used to evaluate the response of the system using a range of different performance metrics that address sustainability, reliability, resilience, and equity. All these metrics will be affected by the interaction of the behaviors and decisions of the different agents; for example, if a short-term component failure or a longer-term drought requires the curtailment of system demand, how will such curtailment be managed (e.g., conservation, increased water rates, etc.), and how will the customers respond (e.g., conserve water, supplement with bottled water, complain to the governing board, relocate, etc.)? Alternatively, if the demand is decreased, this could lead to negative economic impacts for the utility or a decrease in water quality, leading to disproportionate social impacts on parts of the system or a violation of environmental regulations. Thus, by evaluating the impact of the behaviors and decisions of each class of agents to such scenarios, new insights can be obtained, leading to better long-term management policies.

## 2. Traditional and Emerging Uses of Water Distribution Models

Computer models (i.e., hydraulics and water quality) have traditionally been used in the design and operation of water distribution systems for over 70 years [2]. Historically, such models have been applied in an iterative process for developing a design or operational policy for a water distribution system. More recently, emerging approaches such as using these models to develop digital twins of water utilities have evolved to enable off-line real-time modelling or on-line real-time control [3].

In general, such models have normally been focused on the accurate prediction of flows, pressures, and water quality (e.g., chlorine residuals) in response to an explicit set of operational specifications, including prescribed customer demands. However, in each case, such modelling is typically done in isolation from an explicit understanding or even consideration of the many additional operational factors that can directly impact both consumer demand and the overall health and vitality of the water utility. In addition, such modelling is typically done in a steady state environment or an environment focuses on a daily operational time horizon, without considering the cumulative impacts of other operational metrics (e.g., increases of operation and maintenance costs, water rates, component deterioration and failure) on long-term system performance, sustainability, reliability, or resilience. We would now argue that the current state of computer modelling of water distribution systems (including the emergence of sophisticated artificial intelligence tools) needs explicit inclusion of such additional metrics in utility modelling and management.

## 3. Conceptualization of an Integrated Model of Utility Functionality and Performance

**Figure 1.**
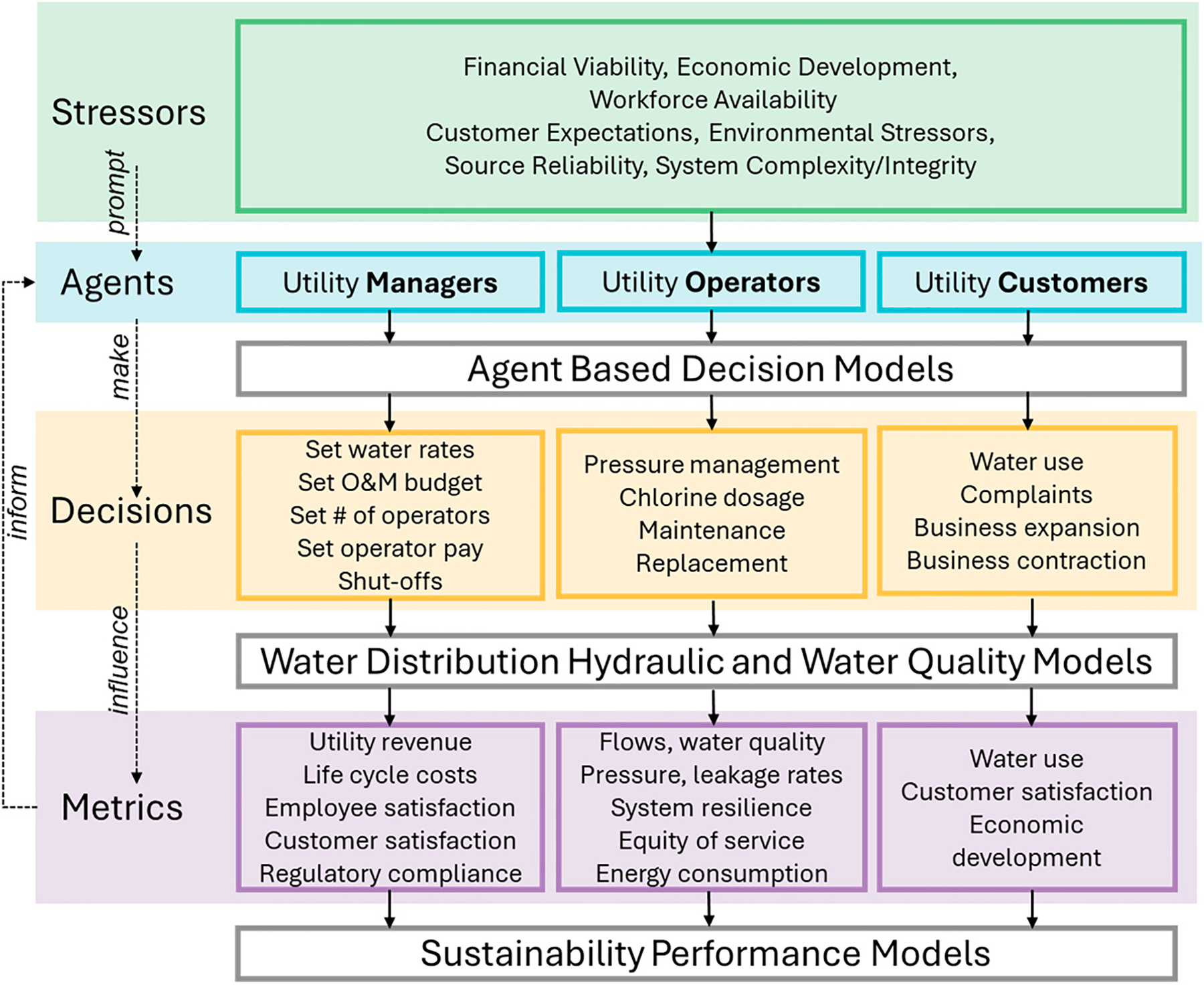
An Agent-Based System Dynamics Model of a Water Utility.

In the proposed approach, we advocate the development of an expanded model of utility functionality that combines a traditional computational model of system hydraulics, energy, and water quality with computational models of agent-based decision-makers and models of sustainability performance (see Figure 1). Currently, we have proposed the development of three different categories of agents (i.e., utility managers, utility operators, and utility customers) that can be treated as single aggregate units (e.g., managers and operators), discretized into different types of subagents (e.g., different types of customers), or individual agents with distinct geographical locations. For example, customers could be treated as different agents based on common characteristics—e.g., the type of water use: residential users, commercial users, industrial users, governmental users; or other racial, social, or economic factors). Alternatively, different customer agents could be discretized and assigned to each service area or demand node in the computational model of the actual water distribution system. All three types of agents and their decisions (e.g., water rates, operational strategies, water use) are then mapped to the computational models of the distribution system through explicit or implicit linkages to system components. For example, operator decisions related to pressure management or water quality can be explicitly mapped to the distribution model using explicit pump settings or disinfection levels. Manager decisions related to operator functionality (e.g., operator resources and motivation) or customer demand (e.g., water rates) can be implicitly linked to the distribution model system components by determining the associated responses of the other agents (i.e., operators or customers). For example, if the manager agent decides to decrease the financial resources to the operators, this may negatively impact the operator’s ability or motivation to repair leaks and breaks promptly, which can be translated into leaving different pipes or components in the network model closed for an extended time, thus impacting system performance and customer satisfaction. If the manager decides to increase the customer water rates, this could lead to a reduction in customer demand, which can then be translated to a decrease in the nodal water demands in the distribution model. This may lead to excessive pressures in the network or a decrease in water quality, which could then lead to a decrease in customer satisfaction through a feedback loop to the customer agents. Such functionality will require the development of different response functions for each agent in response to other agent decisions or other external stressors.

To build sufficient dynamic response capability into the overall model structure, a third model (or group of integrated sub-models) used to measure the overall sustainability of the system will also be required. This model will require the development and quantification of different metrics for measuring sustainability indicators of the overall system. These metrics would be quantified in response to specific agent decisions (connecting to agent-based modelling) as well as the physical infrastructure (connecting to water distribution system modelling). Given the wide variety of potential sustainability indicators (including environmental, economic, and social factors) relevant to this system, this model could be built on the integration of multiple tools, taking advantage of existing approaches such as life cycle assessment (LCA) for quantifying global environmental impacts of the system across its entire lifetime [4].

## 4. Construction of an Integrated Model of Utility Functionality and Performance

Construction of the integrated model requires the development of specific agent-based decision models, calibrated hydraulic and water quality models of the distribution system along with the ability to predict energy consumption of the different components, and a set of models to predict system sustainability in response to both agent-based decisions and physical metrics from the water distribution models. Once assembled, the integrated model can be used to evaluate the impact of individual agent decisions, or in a broader sense, the response of the different agents and the physical distribution system to other external stressors (e.g., financial viability, economic development, or environmental stressors, such as those triggered by climate change, component failures, etc.). The development of such models will require a range of data collection efforts, both for use in calibrating the physical characteristics of the distribution models, but also in constructing and validating both the agent-based decision models and the system sustainability performance models.

Conceptually, the overall structure and framework of the model could be constructed using either a systems dynamic modelling approach or a more traditional agent-based modelling approach. Various system dynamic modelling platforms already exist that could be applied to this problem or spatially explicit agent-based models could be built that would be capable of tracing agents’ behavioral decisions to water distribution and sustainability performance models using some type of structured programming language such as Python. Such resolution is needed to investigate distributional impacts and so called ‘soft adaptation constraints’ arising from behavioral heterogeneity [5]. Once constructed, calibrated, and validated, the model could then be used to generate repeated system responses for different system stressors or agent actions, which could then be synthesized into a more predictive model using methods from the field of artificial intelligence. This predictive model could then be used to explore and identify optimal agent responses to maximize the overall system sustainability in response to specific stressors or other time varying constraints. Given the potential stochastic nature of the agent-based decisions and responses, such variability could be further explored through more traditional Monte Carlo analyses.

## Data Availability

No transferrable data were created as part of this research.
